# Bis(*N*,*N*-diethyl-4-methyl-4-piperazine-1-carboxamide) tetra­kis­(iso­thio­cyanato-κ*N*)­cobalt(II), a model compound for the blue color developed in the Scott test

**DOI:** 10.1107/S2056989023000981

**Published:** 2023-02-09

**Authors:** Allen G. Oliver, Tracy-Lynn E. Lockwood, Jessica Zinna, Marya Lieberman

**Affiliations:** a Department of Chemistry and Biochemistry, University of Notre Dame, Notre Dame, IN 46556, USA; Purdue University, USA

**Keywords:** crystal structure, cobalt(II)iso­thio­cyante, di­ethyl­carbamazine, Scott test

## Abstract

The complex, bis­(*N*,*N*-diethyl-4-methyl-4-piperazine-1-carboxamide) tetra­kis(iso­thio­cyanato-κ*N*)cobalt(II) (*N*,*N*-diethyl-4-methyl-4-piperazine-1-carboxamide = di­ethyl­carbamazine), (C_10_H_22_N_3_O)_2_[Co(NCS)_4_], is presented. This com­plex is a blue precipitate, insoluble in water but soluble in organic solvents, that is formed from the reaction of diethylcarbamazine citrate, a protonated tertiary amine, with cobalt(II) and thio­cyanate. This reaction, in the form of the Scott test, is a common presumptive test for cocaine hydro­chloride. The known cobalt compound, [K_2_Co(NCS)_4_]·3H_2_O, has a deep-blue coloration due to the tetra­hedral [Co(NCS)_4_]^2−^ that is also present in the ion pair with bulky amines, and is similar to the color of other tetra­hedral cobalt(II) complex ions, such as [CoCl_4_]^2−^. The structure is consistent with a previous proposal that a hydro­phobic ion pair formed between [Co(NCS)_4_]^2−^ and two protonated mol­ecules of cocaine is responsible for the blue hydro­phobic products formed by cocaine in the Scott test.

## Abstract 2

The structure of bis­(*N*,*N*-diethyl-4-methyl-4-piperazine-1-carboxamide) tetra­kis­(iso­thio­cyanto)cobalt(II) [or bis­(carbamazide) tetra­kis­(iso­thio­cyanato)­cobalt(II)] is reported. This complex represents an example of the compound found in the colormetric response in Scott’s test.

## 1. Chemical context

In forensics and law enforcement, the Scott test, and modifications to that test (Scott, 1973 ▸; Fansello & Higgins, 1986 ▸; Tsujikawa *et al.*, 2017 ▸), provide identification of tertiary amines from opioids present in a sample. However, there are few reports on the nature of the coloration that is observed during this test, which can vary from powder blue to royal blue, purple, blue–green, or green depending on the identity of the tertiary amine being tested. Oguri and co-workers found that the blue precipitates from cocaine hydro­chloride have a 1:2 cobalt:cocaine stoichiometry (Oguri *et al.*, 1995 ▸), and IR spectra show the blue precipitates contain one or more thio­cyanate units (Morris, 2007 ▸). However, the strong blue color is consistent with a tetra­hedral Co^II^ species, rather than the octa­hedral structure postulated by Oguri and co-workers.

As part of our on-going research into detection of functional groups using Paper Analytical Devices (PADs, Weaver *et al.*, 2013 ▸; idPADs Lockwood *et al.*, 2020 ▸), we sought to understand why tertiary amines give blue precipitates of so many colors in the presence of the Scott reagent. The citrate salt (di­ethyl­carbamazinium citrate; CAS#1642-54-2) of a suitable tertiary amine (di­eth­ylcarbamazine; CAS#90-89-1) was selected as a representative tertiary amine. The title compound was prepared by extraction into a CH_2_Cl_2_ solution from a dried, stoichiometric mixture (1:2) of K_2_[Co(NCS)_4_] and di­ethyl­carbamazinium citrate that yielded the blue crystals used in this study. The tetra­hedral ion [Co(NCS)_4_]^2−^ can also be readily formed by disproportionation of the reagent used for the Scott test [the neutral compound Co(SCN)_2_] in the presence of a suitable amine, as demonstrated in the synthesis for the iso­thio­pendylium tetra­kis­(iso­thio­cyanato)cobalt(II) complex (refcode: QUXKOK, Arunkashi *et al.*, 2010 ▸), which, like our structure, is an ion pair between two protonated amines and [Co(NCS)_4_]^2−^. 
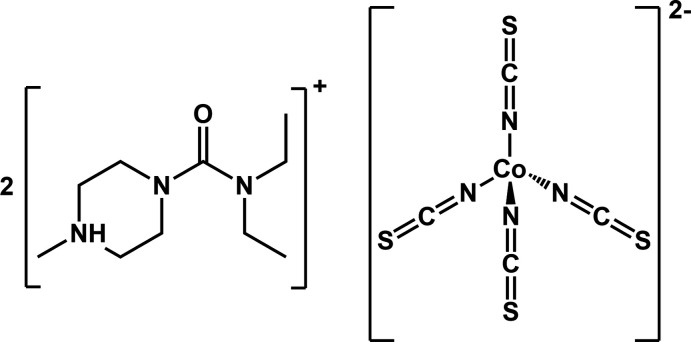


This formulation for the ion pair has been proposed in the Scott test literature for cocaine: [Co^2+^ + 4SCN^−^ +2B: (color red) ←→ [Co(SCN)_4_)B_2_]^2−^ (color blue)] (Conceição *et al.*, 2014 ▸, in Portugese), and the [(cocaineH)_2_[Co(NCS)_4_] ion pair features in several flow-injection analysis methods for cocaine, see for example Eisman *et al.* (1992 ▸). However, there is still no crystal structure for the Scott test product with cocaine, and only three examples are available for protonated tertiary amine ion pairs with the [Co(NCS)_4_]^2−^ dianion.

The Scott test is a three-step sequence of reactions: (1) addition of 2% cobalt thio­cyanate in water; (2) addition of 1.2 *M* HCl solution; (3) addition of chloro­form. We ascribe the initial blue precipitate in the Scott test to the formation of the ion pair (amineH)_2_[Co(NCS)_4_]. Formation of the ion pair should be a reversible reaction, so when concentrated HCl is added in the second step and it protonates the thio­cyanate ions (p*K*_α_ for HNCS is 1.1), the tetra­hedral cobalt anion falls apart and the blue color vanishes. When chloro­form is added in the final step of the Scott test, the hydro­phobic ion pair reforms in the organic solvent, turning it blue.

## 2. Structural commentary

**Figure 1:**
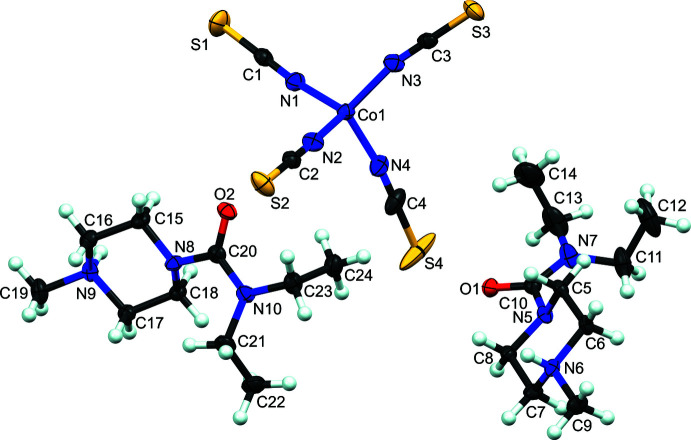
Mol­ecular structure of bis­(di­ethyl­carbamazide) tetra­kis­(iso­thio­cyanato)­cobalt(II). Atomic displacement ellipsoids depicted at the 50% probability level. Hydrogen atoms are shown as spheres of an arbitrary radius.

The complex crystallizes with two protonated di­ethyl­carbamazine cations and one tetra­kis­(iso­thio­cyanato)­cobalt(II) dianion in the asymmetric unit (Fig. 1 ▸). The iso­thio­cyanate ligands are bound to the cobalt through their nitro­gen atoms, leaving the more bulky and hydro­phobic sulfur atoms exposed to the solvent. Protonation of the carbamazines was confirmed by the presence of electron density on the methyl-piperazine nitro­gen atoms N6 and N9. The geometry of the carbamazide mol­ecules is unexceptional. The Co^II^ center adopts a near ideal tetra­hedral geometry (τ_4_ = 0.97; Yang *et al.*, 2007 ▸; Table 1 ▸) that is located in a general position within the asymmetric unit. In contrast, the cobalt center in the parent compound K_2_[Co(NCS)_4_]·3H_2_O is located on a twofold screw-axis (space group *P*2_1_2_1_2; Drew & Othman, 1975 ▸). The τ_4_ metric for the parent compound is 0.94 with the largest N—Co—N angle = 114.1 (3)° [in contrast to 112.10 (7)° reported here]. Although this small change should be considered carefully because the Scott test result is a solution phase analysis and here the solid-state structures are compared, it could indicate that the colorimetric response is a change in the tetra­hedral ligand field about Co.

## 3. Supra­molecular features

**Figure 2:**
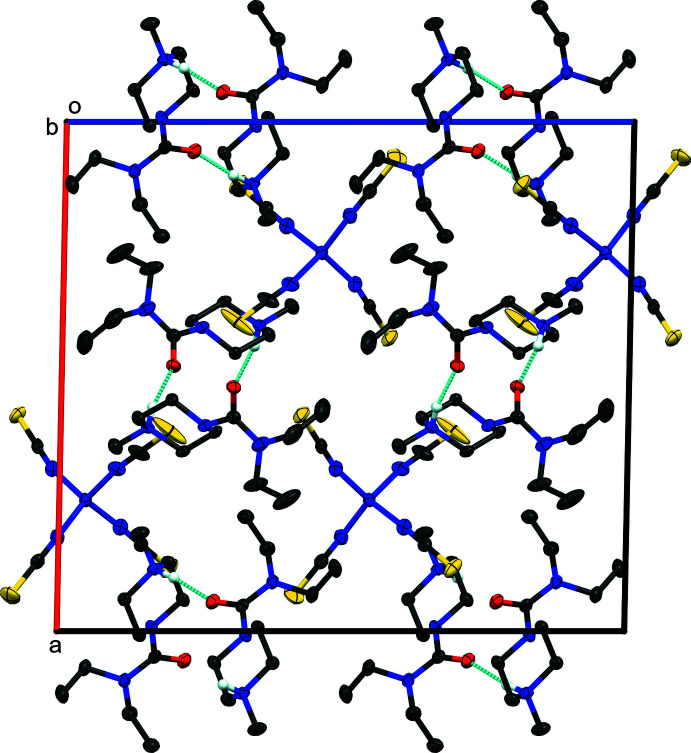
Packing diagram of bis­(di­ethyl­carbamazide) tetra­kis­(iso­thio­cyanato)­cobalt(II) viewed along the *b*-axis. Hydrogen atoms, except for those involved in hydrogen bonding, have been omitted for clarity. Blue dashed lines represent hydrogen-bonding inter­actions.

Both protonated tertiary amine nitro­gen atoms are involved in inter­molecular hydrogen bonding with amide oxygen atoms of an identical mol­ecule related by the screw-axis along the *b*-axis, resulting in mono-periodic chains of each amide along the *b*-axis direction (Fig. 2 ▸). Thus, N6 forms a hydrogen bond to O1^i^ and N9 to O2^ii^ [symmetry codes: (i) −*x* + 1, *y* − 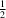 , −*z* + 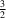 ; (ii) −*x* + 2, *y* + 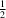 , −*z* + 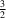 ; see Table 2 ▸ for details]. Both chains are identified as having graph-set motif 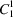 (7) (Etter *et al.*, 1990 ▸).

## 4. Database survey

The core structure of *N*,*N*-diethyl-4-methyl-4-piperazine-1-carboxamide is only reported in five instances in the Cambridge Structural Database (CSD, v 5.43, update 4, November 2022; Groom *et al.*, 2016 ▸). One is a diphenyl morpholine derivative, [4-(di­phenyl­meth­yl)-piperazin-1-yl](morpholin-4-yl)methanone (refcode: IDOVAB, Kumar *et al.*, 2017 ▸). The remaining four reported structures are a series of citrates reported by da Silva and co-workers (refcodes: QURWOQ, QURWOQ01, QURWOQ02, and QURWOQ03; da Silva *et al.*, 2010 ▸). Di­ethyl­carbamazide citrate is used widely in the treatment of filariasis. Comparing the di­ethyl­carbamazide mol­ecules reported herein with those with citrate counter-ions reported by da Silva, the structures are essentially identical. Two of da Silva’s structures have some ethyl chain disorder that is the only significant difference compared with the structure reported here. Tetra­kis(iso­thio­cyanato)­cobalt(II) is reported in over 200 structures. At the inter­section of (iso­thio­cyanto)cobalt and tertiary amines there are five structures. Two of these structures contain hexa­kis­(iso­thio­cyanato)­cobalt (refcode: ILOXEP, Makhlouf, 2021 ▸; KIPYUD, Mali *et al.*, 1991 ▸) and are not pertinent to the discussion. The remaining three compounds {QUXKOK, [*N*,*N*-dimethyl-1-(10*H*-pyrido[3,2-*b*][1,4]benzo­thia­zin-10-yl)propan-2-aminium] (iso­thio­pendylium), Arunkashi *et al.*, 2010 ▸; XIXQUT, [tri­methyl­ammonium], Jie *et al.*, 2018 ▸; YEPHIK, [2-di­ethyl­amino-*N*-(2,6-di­methyl­phen­yl)acetamide] (lignocainium), Qayyas *et al.*, 1994 ▸} contain a tetra­kis­(iso­thio­cyanato)cobalt(II) anion and associated tertiary amine cation. Bond angles about the cobalt centers in these three structures are similar to those reported here (range for angles about Co is 104.78 to 114.05°).

## 5. Synthesis and crystallization

K_2_[Co(NCS)_4_] was prepared by the metathesis of Co(NO_3_)_2_ (3.00 g, 16.4 mmol) and K(SCN) (3.88 g, 39.9 mmol) in 20 mL of water and allowed to dry. Dark-blue crystals were harvested for subsequent reactions; note: upon dissolution in water the solution is pink. K_2_[Co(NCS)_4_] and di­ethyl­carbamazide citrate were mixed in a stoichiometric (1:2) ratio in water and allowed to dry. CHCl_3_ or CH_2_Cl_2_ was added to extract the blue complex. Crystals were grown from the CH_2_Cl_2_ extract by vapor diffusion of hexane at 277 K.

## 6. Refinement

Crystal data, data collection and structure refinement details are summarized in Table 3 ▸. Hydrogen atoms bonded to tertiary amine nitro­gen atoms (N6, N9) were refined freely. All other hydrogen atoms were included in geometrically calculated positions with C—H bond distances constrained to 0.98 Å for aromatic and methyl­ene and 0.99 Å for methyl hydrogen atoms with *U*_iso_(*H*) = 1.5*U*_eq_(C) for methyl and 1.2*U*_eq_(C) for aromatic and methyl­ene H atoms.

## Supplementary Material

Crystal structure: contains datablock(s) I. DOI: 10.1107/S2056989023000981/zl5041sup1.cif

Structure factors: contains datablock(s) I. DOI: 10.1107/S2056989023000981/zl5041Isup2.hkl

CCDC reference: 2239646

Additional supporting information:  crystallographic information; 3D view; checkCIF report

## Figures and Tables

**Table 1:** Selected geometric parameters (Å, °)

Co1—N3	1.9412 (18)	Co1—N1	1.9502 (17)
Co1—N4	1.9451 (18)	Co1—N2	1.9520 (18)
			
N3—Co1—N4	110.91 (8)	N3—Co1—N2	108.49 (7)
N3—Co1—N1	112.10 (7)	N4—Co1—N2	106.51 (8)
N4—Co1—N1	109.98 (8)	N1—Co1—N2	108.66 (8)

**Table 2:** Hydrogen-bond geometry (Å, °)

*D*—H⋯*A*	*D*—H	H⋯*A*	*D*⋯*A*	*D*—H⋯*A*
N6—H6⋯O1^i^	0.84 (2)	1.86 (2)	2.691 (2)	169 (2)
N9—H9⋯O2^ii^	0.88 (2)	1.82 (2)	2.694 (2)	172 (2)

Symmetry codes: (i) 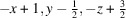 ; (ii) 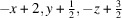 .

**Table 3:** Experimental details

Crystal data	
Chemical formula	(C_10_H_22_N_3_O)_2_[Co(NCS)_4_]
*M*_r_	691.86
Crystal system, space group	Monoclinic, *P*2_1_/*c*
Temperature (K)	120
*a*, *b*, *c* (Å)	17.915 (2), 9.8192 (13), 19.954 (3)
β (°)	91.150 (2)
*V* (Å^3^)	3509.4 (8)
*Z*	4
Radiation type	Mo *K*α
μ (mm^−1^)	0.76
Crystal size (mm)	0.28 × 0.07 × 0.06

Data collection	
Diffractometer	Bruker APEXII
Absorption correction	Numerical (*SADABS*; Krause *et al.*, 2015 ▸)
*T*_min_, *T*_max_	0.810, 0.979
No. of measured, independent and observed [*I* > 2σ(*I*)] reflections	66480, 8799, 6514
*R*_int_	0.052
(sin θ/λ)_max_ (Å^−1^)	0.670

Refinement	
*R*[*F* ^2^ > 2σ(*F* ^2^)], *wR*(*F* ^2^), *S*	0.039, 0.087, 1.01
No. of reflections	8799
No. of parameters	384
H-atom treatment	H atoms treated by a mixture of independent and constrained refinement
Δρ_max_, Δρ_min_ (e Å^−3^)	0.98, −1.11

Computer programs: *APEX3* and *SAINT* (Bruker, 2018 ▸), *SHELXT2014/5* (Sheldrick, 2015*a* ▸), *SHELXL2018/3* (Sheldrick, 2015*b* ▸), *Mercury* (Macrae *et al.*, 2020 ▸), and *publCIF* (Westrip, 2010 ▸).

## supplementary crystallographic information

### Crystal data

**Table:**

(C_10_H_22_N_3_O)_2_[Co(NCS)_4_]	*F*(000) = 1460
*M**_r_* = 691.86	*D*_x_ = 1.309 Mg m^−^^3^
Monoclinic, *P*2_1_/*c*	Mo *K*α radiation, λ = 0.71073 Å
*a* = 17.915 (2) Å	Cell parameters from 9913 reflections
*b* = 9.8192 (13) Å	θ = 2.3–27.6°
*c* = 19.954 (3) Å	µ = 0.76 mm^−^^1^
β = 91.150 (2)°	*T* = 120 K
*V* = 3509.4 (8) Å^3^	Rod, blue
*Z* = 4	0.28 × 0.07 × 0.06 mm

### Data collection

**Table:**

Bruker APEXII diffractometer	8799 independent reflections
Radiation source: fine-focus sealed tube	6514 reflections with *I* > 2σ(*I*)
Detector resolution: 8.33 pixels mm^-1^	*R*_int_ = 0.052
combination of ω and φ–scans	θ_max_ = 28.4°, θ_min_ = 1.1°
Absorption correction: numerical (SADABS; Krause *et al.*, 2015)	*h* = −23→23
*T*_min_ = 0.810, *T*_max_ = 0.979	*k* = −13→13
66480 measured reflections	*l* = −26→26

### Refinement

**Table:**

Refinement on *F*^2^	Primary atom site location: dual
Least-squares matrix: full	Secondary atom site location: difference Fourier map
*R*[*F*^2^ > 2σ(*F*^2^)] = 0.039	Hydrogen site location: mixed
*wR*(*F*^2^) = 0.087	H atoms treated by a mixture of independent and constrained refinement
*S* = 1.01	*w* = 1/[σ^2^(*F*_o_^2^) + (0.030*P*)^2^ + 2.9287*P*] where *P* = (*F*_o_^2^ + 2*F*_c_^2^)/3
8799 reflections	(Δ/σ)_max_ = 0.002
384 parameters	Δρ_max_ = 0.98 e Å^−^^3^
0 restraints	Δρ_min_ = −1.11 e Å^−^^3^

### Special details

**Table:**

Geometry. All esds (except the esd in the dihedral angle between two l.s. planes) are estimated using the full covariance matrix. The cell esds are taken into account individually in the estimation of esds in distances, angles and torsion angles; correlations between esds in cell parameters are only used when they are defined by crystal symmetry. An approximate (isotropic) treatment of cell esds is used for estimating esds involving l.s. planes.

### Fractional atomic coordinates and isotropic or equivalent isotropic displacement parameters (Å^2^)

**Table:**

	*x*	*y*	*z*	*U*_iso_*/*U*_eq_	
Co1	0.74254 (2)	0.61672 (3)	0.54618 (2)	0.02168 (7)	
S1	0.92990 (4)	0.81311 (8)	0.41695 (4)	0.05019 (18)	
S2	0.87654 (4)	0.32929 (6)	0.69618 (3)	0.03974 (15)	
S3	0.57552 (3)	0.33888 (7)	0.42353 (3)	0.03850 (14)	
S4	0.60666 (5)	0.89379 (7)	0.69206 (6)	0.0898 (4)	
N1	0.81550 (10)	0.72430 (18)	0.49788 (8)	0.0287 (4)	
N2	0.79572 (10)	0.49630 (18)	0.60861 (9)	0.0296 (4)	
N3	0.68112 (10)	0.50601 (18)	0.48628 (8)	0.0286 (4)	
N4	0.68106 (10)	0.73551 (18)	0.60020 (9)	0.0313 (4)	
C1	0.86334 (12)	0.7621 (2)	0.46387 (10)	0.0264 (4)	
C2	0.83000 (11)	0.4275 (2)	0.64557 (10)	0.0247 (4)	
C3	0.63674 (12)	0.4369 (2)	0.45962 (9)	0.0249 (4)	
C4	0.64989 (13)	0.8006 (2)	0.63879 (13)	0.0373 (6)	
O1	0.47917 (7)	1.15381 (14)	0.69673 (7)	0.0262 (3)	
N5	0.40377 (9)	0.99660 (16)	0.74649 (8)	0.0205 (3)	
N6	0.39973 (9)	0.78750 (17)	0.84633 (8)	0.0209 (3)	
H6	0.4399 (12)	0.747 (2)	0.8383 (10)	0.022 (6)*	
N7	0.36711 (10)	1.10644 (18)	0.64724 (9)	0.0324 (4)	
C5	0.39077 (11)	0.85401 (18)	0.72708 (9)	0.0210 (4)	
H5A	0.438946	0.809565	0.717291	0.025*	
H5B	0.358850	0.850411	0.686039	0.025*	
C6	0.35303 (10)	0.7795 (2)	0.78338 (9)	0.0222 (4)	
H6A	0.303459	0.820433	0.791130	0.027*	
H6B	0.345499	0.682859	0.770736	0.027*	
C7	0.41778 (11)	0.9324 (2)	0.86332 (10)	0.0241 (4)	
H7A	0.451831	0.935096	0.902995	0.029*	
H7B	0.371368	0.981171	0.874750	0.029*	
C8	0.45436 (11)	1.0032 (2)	0.80495 (9)	0.0216 (4)	
H8A	0.464916	1.099404	0.816562	0.026*	
H8B	0.502182	0.957921	0.794761	0.026*	
C9	0.36244 (12)	0.7175 (2)	0.90305 (10)	0.0304 (5)	
H9A	0.393790	0.725334	0.943679	0.046*	
H9B	0.355143	0.621148	0.891989	0.046*	
H9C	0.313896	0.760209	0.910702	0.046*	
C10	0.41985 (11)	1.08939 (19)	0.69569 (10)	0.0223 (4)	
C11	0.28776 (13)	1.0721 (3)	0.65439 (14)	0.0458 (6)	
H11A	0.258149	1.157241	0.654449	0.055*	
H11B	0.280949	1.026491	0.698088	0.055*	
C12	0.25841 (16)	0.9804 (3)	0.59917 (17)	0.0664 (9)	
H12A	0.260202	1.028600	0.556220	0.100*	
H12B	0.206713	0.954935	0.608292	0.100*	
H12C	0.289270	0.898163	0.597135	0.100*	
C13	0.38615 (16)	1.1942 (3)	0.59024 (14)	0.0519 (7)	
H13A	0.413605	1.275130	0.607109	0.062*	
H13B	0.339571	1.226213	0.567837	0.062*	
C14	0.43331 (19)	1.1209 (4)	0.53983 (14)	0.0682 (9)	
H14A	0.446433	1.183855	0.503827	0.102*	
H14B	0.405125	1.043954	0.520909	0.102*	
H14C	0.478997	1.087235	0.562009	0.102*	
O2	0.94210 (8)	0.06408 (14)	0.77411 (7)	0.0263 (3)	
N8	1.00777 (9)	0.22499 (16)	0.83271 (8)	0.0212 (3)	
N9	1.12181 (9)	0.42920 (17)	0.82969 (9)	0.0230 (3)	
H9	1.1052 (12)	0.478 (2)	0.7954 (11)	0.034 (6)*	
N10	0.90345 (9)	0.11786 (17)	0.87878 (8)	0.0240 (3)	
C15	1.06216 (11)	0.2245 (2)	0.77881 (10)	0.0249 (4)	
H15A	1.042531	0.277347	0.740055	0.030*	
H15B	1.071278	0.129933	0.763850	0.030*	
C16	1.13451 (11)	0.2875 (2)	0.80479 (10)	0.0261 (4)	
H16A	1.155428	0.230933	0.841675	0.031*	
H16B	1.171222	0.289546	0.768375	0.031*	
C17	1.06253 (10)	0.4300 (2)	0.88160 (9)	0.0229 (4)	
H17A	1.051975	0.525004	0.895086	0.028*	
H17B	1.080422	0.379663	0.921790	0.028*	
C18	0.99185 (10)	0.36465 (18)	0.85434 (9)	0.0209 (4)	
H18A	0.953590	0.363374	0.889451	0.025*	
H18B	0.972047	0.418252	0.815905	0.025*	
C19	1.19218 (11)	0.4913 (2)	0.85676 (12)	0.0329 (5)	
H19A	1.183296	0.587218	0.867644	0.049*	
H19B	1.231081	0.485059	0.823061	0.049*	
H19C	1.208379	0.442525	0.897345	0.049*	
C20	0.94966 (10)	0.13172 (18)	0.82610 (9)	0.0209 (4)	
C21	0.92829 (12)	0.1378 (2)	0.94905 (10)	0.0303 (5)	
H21A	0.978919	0.178300	0.949711	0.036*	
H21B	0.931594	0.048027	0.971455	0.036*	
C22	0.87648 (14)	0.2293 (3)	0.98833 (11)	0.0408 (6)	
H22A	0.876818	0.321136	0.969135	0.061*	
H22B	0.893574	0.233173	1.035258	0.061*	
H22C	0.825651	0.192474	0.985933	0.061*	
C23	0.83827 (11)	0.0288 (2)	0.86836 (11)	0.0291 (4)	
H23A	0.819629	0.000020	0.912537	0.035*	
H23B	0.853961	−0.054001	0.844096	0.035*	
C24	0.77536 (12)	0.0968 (2)	0.82916 (11)	0.0334 (5)	
H24A	0.793927	0.129085	0.786114	0.050*	
H24B	0.756463	0.174142	0.854792	0.050*	
H24C	0.734967	0.031080	0.821181	0.050*	

### Atomic displacement parameters (Å^2^)

**Table:**

	*U*^11^	*U*^22^	*U*^33^	*U*^12^	*U*^13^	*U*^23^
Co1	0.02440 (13)	0.02027 (13)	0.02043 (13)	−0.00148 (11)	0.00194 (10)	0.00012 (11)
S1	0.0401 (4)	0.0601 (4)	0.0510 (4)	−0.0233 (3)	0.0147 (3)	0.0044 (3)
S2	0.0480 (4)	0.0311 (3)	0.0393 (3)	−0.0055 (3)	−0.0194 (3)	0.0092 (2)
S3	0.0379 (3)	0.0448 (3)	0.0323 (3)	−0.0053 (3)	−0.0132 (2)	−0.0080 (3)
S4	0.0949 (6)	0.0321 (4)	0.1462 (9)	−0.0231 (4)	0.0978 (7)	−0.0344 (5)
N1	0.0318 (10)	0.0283 (9)	0.0260 (9)	−0.0050 (8)	0.0033 (7)	0.0005 (7)
N2	0.0343 (10)	0.0263 (9)	0.0282 (9)	0.0004 (8)	−0.0015 (8)	−0.0002 (7)
N3	0.0345 (10)	0.0271 (9)	0.0242 (9)	−0.0035 (8)	−0.0002 (7)	0.0006 (7)
N4	0.0283 (9)	0.0266 (9)	0.0393 (10)	−0.0023 (8)	0.0067 (8)	−0.0045 (8)
C1	0.0304 (11)	0.0226 (10)	0.0261 (10)	−0.0058 (8)	−0.0033 (9)	−0.0026 (8)
C2	0.0282 (10)	0.0213 (10)	0.0247 (10)	−0.0065 (8)	−0.0007 (8)	−0.0027 (8)
C3	0.0307 (11)	0.0263 (10)	0.0177 (9)	0.0046 (9)	0.0005 (8)	0.0025 (8)
C4	0.0333 (12)	0.0190 (10)	0.0604 (15)	−0.0071 (9)	0.0214 (11)	−0.0025 (10)
O1	0.0246 (7)	0.0281 (7)	0.0261 (7)	−0.0076 (6)	0.0031 (6)	0.0000 (6)
N5	0.0212 (8)	0.0165 (8)	0.0238 (8)	−0.0024 (6)	−0.0023 (6)	−0.0015 (6)
N6	0.0189 (8)	0.0209 (8)	0.0229 (8)	0.0017 (7)	0.0025 (6)	−0.0008 (6)
N7	0.0294 (9)	0.0268 (9)	0.0406 (10)	−0.0038 (8)	−0.0102 (8)	0.0111 (8)
C5	0.0225 (9)	0.0174 (9)	0.0229 (9)	−0.0010 (7)	−0.0062 (7)	−0.0025 (7)
C6	0.0188 (9)	0.0196 (9)	0.0281 (10)	−0.0024 (7)	−0.0046 (8)	0.0006 (8)
C7	0.0284 (10)	0.0205 (9)	0.0236 (10)	−0.0014 (8)	0.0044 (8)	−0.0047 (8)
C8	0.0221 (9)	0.0210 (9)	0.0216 (9)	−0.0037 (8)	0.0004 (7)	−0.0038 (7)
C9	0.0344 (12)	0.0283 (11)	0.0287 (11)	−0.0033 (9)	0.0071 (9)	0.0054 (9)
C10	0.0232 (10)	0.0176 (9)	0.0261 (10)	0.0009 (7)	0.0010 (8)	−0.0018 (7)
C11	0.0265 (12)	0.0362 (13)	0.0738 (18)	−0.0012 (10)	−0.0165 (12)	0.0140 (13)
C12	0.0529 (17)	0.0469 (17)	0.097 (2)	−0.0115 (14)	−0.0454 (17)	0.0176 (16)
C13	0.0565 (17)	0.0458 (16)	0.0523 (16)	−0.0158 (13)	−0.0244 (13)	0.0284 (13)
C14	0.076 (2)	0.094 (3)	0.0339 (14)	−0.0269 (19)	−0.0073 (14)	0.0207 (16)
O2	0.0295 (7)	0.0231 (7)	0.0260 (7)	0.0027 (6)	−0.0047 (6)	−0.0068 (6)
N8	0.0246 (8)	0.0180 (8)	0.0212 (8)	−0.0009 (6)	0.0046 (6)	−0.0029 (6)
N9	0.0203 (8)	0.0206 (8)	0.0280 (9)	0.0013 (7)	−0.0026 (7)	0.0067 (7)
N10	0.0270 (8)	0.0249 (8)	0.0201 (8)	−0.0042 (7)	−0.0019 (6)	0.0050 (7)
C15	0.0279 (10)	0.0222 (10)	0.0250 (10)	0.0027 (8)	0.0060 (8)	−0.0032 (8)
C16	0.0244 (10)	0.0238 (10)	0.0304 (11)	0.0050 (8)	0.0039 (8)	0.0006 (8)
C17	0.0244 (10)	0.0211 (9)	0.0233 (9)	0.0021 (8)	−0.0004 (8)	−0.0011 (8)
C18	0.0227 (9)	0.0177 (9)	0.0225 (9)	0.0019 (7)	0.0018 (7)	−0.0005 (7)
C19	0.0230 (10)	0.0282 (11)	0.0472 (13)	−0.0028 (9)	−0.0060 (9)	0.0044 (10)
C20	0.0245 (9)	0.0157 (9)	0.0225 (9)	0.0045 (7)	−0.0031 (7)	0.0040 (7)
C21	0.0360 (12)	0.0347 (12)	0.0201 (10)	−0.0050 (9)	−0.0039 (8)	0.0082 (8)
C22	0.0464 (14)	0.0538 (15)	0.0227 (11)	−0.0084 (12)	0.0076 (10)	0.0005 (10)
C23	0.0290 (11)	0.0260 (11)	0.0322 (11)	−0.0061 (9)	−0.0015 (9)	0.0072 (9)
C24	0.0257 (11)	0.0375 (13)	0.0369 (12)	−0.0022 (9)	0.0011 (9)	0.0055 (10)

### Geometric parameters (Å, º)

**Table:**

Co1—N3	1.9412 (18)	C13—C14	1.509 (4)
Co1—N4	1.9451 (18)	C13—H13A	0.9900
Co1—N1	1.9502 (17)	C13—H13B	0.9900
Co1—N2	1.9520 (18)	C14—H14A	0.9800
S1—C1	1.611 (2)	C14—H14B	0.9800
S2—C2	1.616 (2)	C14—H14C	0.9800
S3—C3	1.618 (2)	O2—C20	1.237 (2)
S4—C4	1.613 (2)	N8—C20	1.391 (2)
N1—C1	1.165 (3)	N8—C15	1.466 (2)
N2—C2	1.166 (3)	N8—C18	1.467 (2)
N3—C3	1.165 (3)	N9—C19	1.492 (3)
N4—C4	1.153 (3)	N9—C16	1.496 (3)
O1—C10	1.236 (2)	N9—C17	1.498 (2)
N5—C10	1.397 (2)	N9—H9	0.88 (2)
N5—C8	1.464 (2)	N10—C20	1.358 (2)
N5—C5	1.470 (2)	N10—C23	1.470 (3)
N6—C9	1.493 (2)	N10—C21	1.476 (2)
N6—C7	1.497 (2)	C15—C16	1.518 (3)
N6—C6	1.497 (2)	C15—H15A	0.9900
N6—H6	0.84 (2)	C15—H15B	0.9900
N7—C10	1.349 (3)	C16—H16A	0.9900
N7—C11	1.471 (3)	C16—H16B	0.9900
N7—C13	1.472 (3)	C17—C18	1.511 (3)
C5—C6	1.512 (3)	C17—H17A	0.9900
C5—H5A	0.9900	C17—H17B	0.9900
C5—H5B	0.9900	C18—H18A	0.9900
C6—H6A	0.9900	C18—H18B	0.9900
C6—H6B	0.9900	C19—H19A	0.9800
C7—C8	1.516 (3)	C19—H19B	0.9800
C7—H7A	0.9900	C19—H19C	0.9800
C7—H7B	0.9900	C21—C22	1.520 (3)
C8—H8A	0.9900	C21—H21A	0.9900
C8—H8B	0.9900	C21—H21B	0.9900
C9—H9A	0.9800	C22—H22A	0.9800
C9—H9B	0.9800	C22—H22B	0.9800
C9—H9C	0.9800	C22—H22C	0.9800
C11—C12	1.509 (4)	C23—C24	1.514 (3)
C11—H11A	0.9900	C23—H23A	0.9900
C11—H11B	0.9900	C23—H23B	0.9900
C12—H12A	0.9800	C24—H24A	0.9800
C12—H12B	0.9800	C24—H24B	0.9800
C12—H12C	0.9800	C24—H24C	0.9800
			
N3—Co1—N4	110.91 (8)	H13A—C13—H13B	107.9
N3—Co1—N1	112.10 (7)	C13—C14—H14A	109.5
N4—Co1—N1	109.98 (8)	C13—C14—H14B	109.5
N3—Co1—N2	108.49 (7)	H14A—C14—H14B	109.5
N4—Co1—N2	106.51 (8)	C13—C14—H14C	109.5
N1—Co1—N2	108.66 (8)	H14A—C14—H14C	109.5
C1—N1—Co1	165.66 (17)	H14B—C14—H14C	109.5
C2—N2—Co1	177.29 (17)	C20—N8—C15	115.83 (15)
C3—N3—Co1	168.53 (16)	C20—N8—C18	119.59 (15)
C4—N4—Co1	171.6 (2)	C15—N8—C18	110.79 (15)
N1—C1—S1	179.5 (2)	C19—N9—C16	111.56 (16)
N2—C2—S2	178.74 (19)	C19—N9—C17	110.67 (16)
N3—C3—S3	179.0 (2)	C16—N9—C17	110.44 (15)
N4—C4—S4	179.1 (2)	C19—N9—H9	109.3 (15)
C10—N5—C8	114.57 (15)	C16—N9—H9	107.2 (15)
C10—N5—C5	117.62 (15)	C17—N9—H9	107.5 (15)
C8—N5—C5	110.14 (14)	C20—N10—C23	116.42 (16)
C9—N6—C7	111.33 (15)	C20—N10—C21	123.05 (16)
C9—N6—C6	111.14 (15)	C23—N10—C21	115.91 (16)
C7—N6—C6	110.78 (15)	N8—C15—C16	108.90 (16)
C9—N6—H6	108.9 (14)	N8—C15—H15A	109.9
C7—N6—H6	108.3 (15)	C16—C15—H15A	109.9
C6—N6—H6	106.3 (14)	N8—C15—H15B	109.9
C10—N7—C11	124.52 (19)	C16—C15—H15B	109.9
C10—N7—C13	117.16 (18)	H15A—C15—H15B	108.3
C11—N7—C13	116.64 (19)	N9—C16—C15	110.97 (16)
N5—C5—C6	109.64 (15)	N9—C16—H16A	109.4
N5—C5—H5A	109.7	C15—C16—H16A	109.4
C6—C5—H5A	109.7	N9—C16—H16B	109.4
N5—C5—H5B	109.7	C15—C16—H16B	109.4
C6—C5—H5B	109.7	H16A—C16—H16B	108.0
H5A—C5—H5B	108.2	N9—C17—C18	110.44 (15)
N6—C6—C5	110.31 (15)	N9—C17—H17A	109.6
N6—C6—H6A	109.6	C18—C17—H17A	109.6
C5—C6—H6A	109.6	N9—C17—H17B	109.6
N6—C6—H6B	109.6	C18—C17—H17B	109.6
C5—C6—H6B	109.6	H17A—C17—H17B	108.1
H6A—C6—H6B	108.1	N8—C18—C17	109.66 (15)
N6—C7—C8	110.88 (15)	N8—C18—H18A	109.7
N6—C7—H7A	109.5	C17—C18—H18A	109.7
C8—C7—H7A	109.5	N8—C18—H18B	109.7
N6—C7—H7B	109.5	C17—C18—H18B	109.7
C8—C7—H7B	109.5	H18A—C18—H18B	108.2
H7A—C7—H7B	108.1	N9—C19—H19A	109.5
N5—C8—C7	108.76 (15)	N9—C19—H19B	109.5
N5—C8—H8A	109.9	H19A—C19—H19B	109.5
C7—C8—H8A	109.9	N9—C19—H19C	109.5
N5—C8—H8B	109.9	H19A—C19—H19C	109.5
C7—C8—H8B	109.9	H19B—C19—H19C	109.5
H8A—C8—H8B	108.3	O2—C20—N10	122.49 (18)
N6—C9—H9A	109.5	O2—C20—N8	120.16 (17)
N6—C9—H9B	109.5	N10—C20—N8	117.35 (16)
H9A—C9—H9B	109.5	N10—C21—C22	113.13 (18)
N6—C9—H9C	109.5	N10—C21—H21A	109.0
H9A—C9—H9C	109.5	C22—C21—H21A	109.0
H9B—C9—H9C	109.5	N10—C21—H21B	109.0
O1—C10—N7	122.55 (18)	C22—C21—H21B	109.0
O1—C10—N5	120.73 (17)	H21A—C21—H21B	107.8
N7—C10—N5	116.70 (17)	C21—C22—H22A	109.5
N7—C11—C12	112.9 (2)	C21—C22—H22B	109.5
N7—C11—H11A	109.0	H22A—C22—H22B	109.5
C12—C11—H11A	109.0	C21—C22—H22C	109.5
N7—C11—H11B	109.0	H22A—C22—H22C	109.5
C12—C11—H11B	109.0	H22B—C22—H22C	109.5
H11A—C11—H11B	107.8	N10—C23—C24	113.06 (17)
C11—C12—H12A	109.5	N10—C23—H23A	109.0
C11—C12—H12B	109.5	C24—C23—H23A	109.0
H12A—C12—H12B	109.5	N10—C23—H23B	109.0
C11—C12—H12C	109.5	C24—C23—H23B	109.0
H12A—C12—H12C	109.5	H23A—C23—H23B	107.8
H12B—C12—H12C	109.5	C23—C24—H24A	109.5
N7—C13—C14	112.2 (2)	C23—C24—H24B	109.5
N7—C13—H13A	109.2	H24A—C24—H24B	109.5
C14—C13—H13A	109.2	C23—C24—H24C	109.5
N7—C13—H13B	109.2	H24A—C24—H24C	109.5
C14—C13—H13B	109.2	H24B—C24—H24C	109.5
			
C10—N5—C5—C6	−163.66 (16)	C20—N8—C15—C16	−158.44 (16)
C8—N5—C5—C6	62.57 (19)	C18—N8—C15—C16	61.2 (2)
C9—N6—C6—C5	178.50 (16)	C19—N9—C16—C15	178.76 (17)
C7—N6—C6—C5	54.2 (2)	C17—N9—C16—C15	55.2 (2)
N5—C5—C6—N6	−57.8 (2)	N8—C15—C16—N9	−57.8 (2)
C9—N6—C7—C8	−178.98 (16)	C19—N9—C17—C18	−178.96 (16)
C6—N6—C7—C8	−54.8 (2)	C16—N9—C17—C18	−54.9 (2)
C10—N5—C8—C7	162.42 (16)	C20—N8—C18—C17	159.67 (16)
C5—N5—C8—C7	−62.29 (19)	C15—N8—C18—C17	−61.6 (2)
N6—C7—C8—N5	58.3 (2)	N9—C17—C18—N8	57.8 (2)
C11—N7—C10—O1	−157.7 (2)	C23—N10—C20—O2	−6.6 (3)
C13—N7—C10—O1	7.0 (3)	C21—N10—C20—O2	148.25 (19)
C11—N7—C10—N5	20.8 (3)	C23—N10—C20—N8	174.26 (16)
C13—N7—C10—N5	−174.5 (2)	C21—N10—C20—N8	−30.9 (3)
C8—N5—C10—O1	11.3 (2)	C15—N8—C20—O2	−6.9 (3)
C5—N5—C10—O1	−120.47 (19)	C18—N8—C20—O2	129.85 (18)
C8—N5—C10—N7	−167.22 (17)	C15—N8—C20—N10	172.25 (16)
C5—N5—C10—N7	61.0 (2)	C18—N8—C20—N10	−51.0 (2)
C10—N7—C11—C12	−127.1 (2)	C20—N10—C21—C22	131.8 (2)
C13—N7—C11—C12	68.1 (3)	C23—N10—C21—C22	−73.2 (2)
C10—N7—C13—C14	79.7 (3)	C20—N10—C23—C24	−78.8 (2)
C11—N7—C13—C14	−114.4 (3)	C21—N10—C23—C24	124.5 (2)

### Hydrogen-bond geometry (Å, º)

**Table:**

*D*—H···*A*	*D*—H	H···*A*	*D*···*A*	*D*—H···*A*
N6—H6···O1^i^	0.84 (2)	1.86 (2)	2.691 (2)	169 (2)
N9—H9···O2^ii^	0.88 (2)	1.82 (2)	2.694 (2)	172 (2)

Symmetry codes: (i) −*x*+1, *y*−1/2, −*z*+3/2; (ii) −*x*+2, *y*+1/2, −*z*+3/2.
